# 3D-Printed Demineralized Bone Matrix-Based Conductive Scaffolds Combined with Electrical Stimulation for Bone Tissue Engineering Applications

**DOI:** 10.1021/acsabm.4c00236

**Published:** 2024-06-21

**Authors:** Damion T. Dixon, Erika N. Landree, Cheryl T. Gomillion

**Affiliations:** † School of Environmental, Civil, Agricultural and Mechanical Engineering, College of Engineering, University of Georgia, Athens, Georgia 30602, United States; ‡ School of Chemical, Materials and Biomedical Engineering, College of Engineering, University of Georgia, Athens, Georgia 30602, United States

**Keywords:** demineralized bone matrix, electrical stimulation, conductive bone scaffolds, 3D printing, osteogenic differentiation, bone tissue engineering

## Abstract

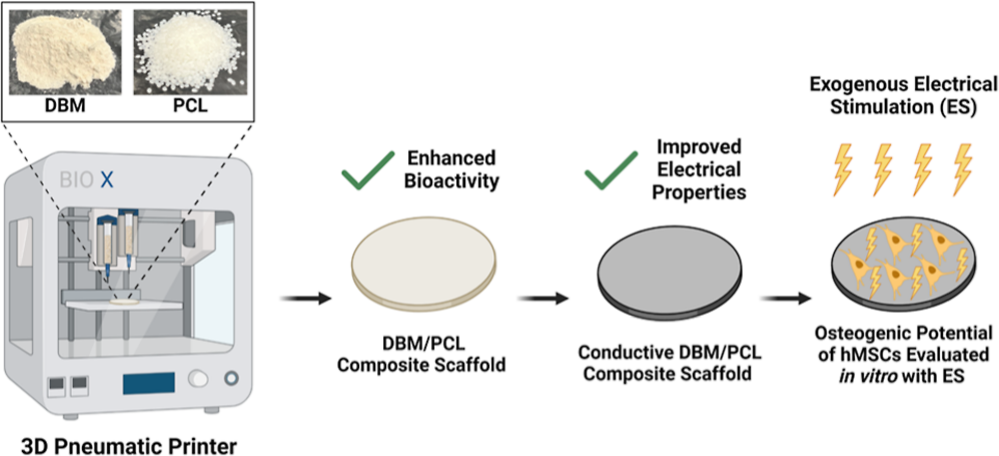

Bone is remodeled through a dynamic process facilitated by biophysical cues that support cellular signaling. In healthy bone, signaling pathways are regulated by cells and the extracellular matrix and transmitted via electrical synapses. To this end, combining electrical stimulation (ES) with conductive scaffolding is a promising approach for repairing damaged bone tissue. Therefore, “smart” biomaterials that can provide multifunctionality and facilitate the transfer of electrical cues directly to cells have become increasingly more studied in bone tissue engineering. Herein, 3D-printed electrically conductive composite scaffolds consisting of demineralized bone matrix (DBM) and polycaprolactone (PCL), in combination with ES, for bone regeneration were evaluated for the first time. The conductive composite scaffolds were fabricated and characterized by evaluating mechanical, surface, and electrical properties. The DBM/PCL composites exhibited a higher compressive modulus (107.2 MPa) than that of pristine PCL (62.02 MPa), as well as improved surface properties (i.e., roughness). Scaffold electrical properties were also tuned, with sheet resistance values as low as 4.77 × 10^5^ Ω/sq for our experimental coating of the highest dilution (i.e., 20%). Furthermore, the biocompatibility and osteogenic potential of the conductive composite scaffolds were tested using human mesenchymal stromal cells (hMSCs) both with and without exogenous ES (100 mV/mm for 5 min/day four times/week). In conjunction with ES, the osteogenic differentiation of hMSCs grown on conductive DBM/PCL composite scaffolds was significantly enhanced when compared to those cultured on PCL-only and nonconductive DBM/PCL control scaffolds, as determined through xylenol orange mineral staining and osteogenic protein analysis. Overall, these promising results suggest the potential of this approach for the development of biomimetic hybrid scaffolds for bone tissue engineering applications.

## 1 Introduction

Bone is a mineralized collagenous tissue and provides structural support for the body as the main component of the skeletal system.^1^ This highly dynamic organ responds to the mechanical demands placed on it, continuously remodeling itself in order to remain structurally sound.^2^ Due to this self-healing ability, minor defects in bone can be repaired without surgical intervention. However, in cases of extensive bone loss due to injury or disease, this self-healing mechanism does not work for large void reconstruction.^3^ Today, autografts serve as the clinical gold standard for repairing bone defects but are limited due to tissue availability, high associated costs, and donor site morbidity.^4,5^ Allografts are the second most common grafting method for repairing bone. These donor-supplied tissues, usually sourced from cadavers, are available in various forms, including demineralized bone matrix (DBM), bone chips, and whole bone segments, dependent on the requirements of the host site. Nonetheless, when compared to autografts, allografts have reduced osteoinductive properties, present a risk of immune rejection and disease transmission, and have no cellular component because of their processing and storage needs.^6^ Due to the shortcomings of natural tissue grafts, regenerative and tissue engineering strategies involving a combination of biocompatible synthetic scaffolds, growth factors, and osteogenic cells have emerged as a promising approach for repairing damaged bone tissue.^7,8^

Bone is a well-studied composite material containing an inorganic matrix of hydroxyapatite and an organic matrix of collagen Type I, approximately 70 and 30% by weight, respectively.^9,10^ To this end, the repair of bone defects using synthetic biomaterials has been a major challenge due to the complex characteristics required to regenerate bone tissue. Biomimetic scaffolds can be fabricated using biodegradable polymers such as polycaprolactone (PCL) and polylactide (PLA); however, such biodegradable polyester materials typically have surface properties incompatible with biological tissues, thus limiting their usefulness as standalone replacements.^11^ Through the rapid development of 3D printing technology, synthetic scaffolds can be designed to mimic the native extracellular matrix. Additionally, enhanced bone regeneration potential can be achieved by including cells and different growth factors, such as bone morphogenetic protein-2 (BMP-2), typically incorporated through covalent binding or adsorption into the biomaterial. Yet, the implantation of these relatively inert polymer-based scaffolds in vivo can be disruptive to native tissue signaling, and the numerous clinical challenges presented by free-releasing growth factors persist.^12^

In bone, collagen serves as the matrix for cell growth, while the inorganic apatite phase serves to supply the mechanical strength for the tissue.^13^ In addition to the previously mentioned characteristics, the piezoelectric potential in bone, the ability of this tissue to convert mechanical stresses into electrical currents, is prompted by these two materials. Applied stresses create local potential gradients along collagen fibers that cause the surrounding particles to become charged, stimulating bone-forming osteoblasts.^14,15^ After large-volume bone loss, this endogenous signaling is compromised; however, with fracture healing, the diminished bioelectric potential at the injury site returns to normal.^16,17^ With this in mind, the combination of exogenous electrical stimulation (ES) and conductive scaffolding is a favorable approach for accelerating the bone regeneration process, replicating endogenous signaling and facilitating the transfer of those signals to growing cells.^18,19^ Therefore, “smart” biomaterials with enhanced biological and electrical properties have become increasingly more studied in bone tissue engineering.^20^

As previously stated, the biological activity of synthetic scaffolds can be improved with the incorporation of growth factors, but their clinical use can be limited due to issues related to spontaneous and uncontrollable release in vivo. As an alternative, allograft DBM is a suitable scaffolding component to improve the biological and structural properties of the typical thermoplastics used for 3D printing synthetic bone. DBM comprises a collagen Type I network containing essential bone-related growth factors like BMPs, which remain after the removal of both the mineralized and the cellular components of bone tissue.^21,22^ As opposed to freely released growth factors, incorporating DBM directly into the scaffolding matrix could provide necessary signaling molecules for supporting bone formation, while potentially improving the mechanical properties of the polymers (e.g., PLA and PCL) commonly used in bone tissue engineering. A wide range of conductive materials have been investigated to produce scaffolds with increased electrical properties, namely, conductive polymers. These organic materials possess electrical and magnetic properties, combining the electroconductivity of metals and semiconductors with the flexibility and ease of the processing found in polymers.^23^ Currently, polypyrrole (PPy), polyaniline (PANI), and poly(3,4-ethylenedioxythiophene) (PEDOT) are among the most promising conductive polymers for scaffold fabrication.^24,25^ These polymers are usually combined with nonconductive biocompatible and biodegradable polymers (e.g., PCL), yet challenges remain due to manufacturing limitations, toxicity concerns, and poor solubility in certain solvents.^26−28^

In this work, we describe methods for developing and validating a printable bioactive composite scaffold capable of supporting both allograft tissues and the electrical signaling necessary for the physiological processes of bone repair to occur. First, using a pneumatic 3D printing technique at ambient temperatures, bioactive DBM/PCL composite scaffolds were fabricated with consistent geometries (circular, thin-film). Then, using a simple two-step polydopamine (PDA)-mediated coating strategy, an optimized conductive coating of poly(3,4-ethylenedioxythiophene):poly(styrenesulfonate) (PEDOT/PPS) and PPy was grafted onto the surface of the composite scaffolds to enhance their electrical properties. The scaffolds were systematically characterized before being analyzed in vitro to assess the effects of different scaffolding combinations (i.e., PCL, DBM/PCL, and conductive DBM/PCL) on cell adhesion, proliferation, and, in combination with ES, osteogenic differentiation. The promising results of this study suggest the potential of this approach for the development of biomimetic hybrid scaffolds for bone tissue engineering applications.

## 2 Materials and Methods

### 2.1 Materials

PCL pellets (*M*_n_ = 80,000), PEDOT/PPS (dry redispersible pellets), PPy (pressed pellets, conductivity = 10–50 S/cm), dopamine hydrochloride, ascorbic acid, dexamethasone, and beta-glycerophosphate disodium salt hydrate (β-GP) were purchased from Sigma-Aldrich (St. Louis, MO, USA). Ethanol (KOPTEC, 200 proof), methanol, and dichloromethane (DCM) were purchased from VWR (Radnor, PA, USA). Chloroform (99.8%, ACROS Organics) and hydrochloric acid (HCl, 36.5 to 38.0%, certified ACS) were purchased from Fisher Scientific (Waltham, MA, USA). Normal (nondiabetic) human bone marrow-derived mesenchymal stromal cells [human mesenchymal stromal cells (hMSCs)] were purchased from Lonza (Basel, Switzerland). Trypsin–EDTA (0.05%), Dulbecco’s phosphate buffered saline (PBS, -Ca, -Mg), penicillin–streptomycin (Pen–Strep), and both low-glucose (1 g/L) and high-glucose (4.5 g/L) Dulbecco’s modified Eagle’s medium (DMEM) were purchased from Gibco (Waltham, MA, USA). Fetal bovine serum (FBS) was purchased from R & D Systems (Minneapolis, MN, USA). Ultra-Low Attachment 24-well culture plates were purchased from Corning (Corning, NY, USA). Platinum wire (25 gauge) was purchased from Thomas Scientific (Swedesboro, NJ, USA). A programmable direct current (DC) power supplier (i.e., B&K Precision Supply-Model 1785B) was purchased from Cole-Parmer (Vernon Hills, IL, USA). The deionized Milli-Q (DI) water (18 MΩ) used in the study was obtained from an in-house purification system.

### 2.2 Printing Ink Formulation, Composite Scaffold Fabrication, and Conductive Coating Process

Human femurs were graciously provided by MTF Biologics (Edison, NJ, USA) through their Non-Transplantable Tissue Program and used to create the DBM powder required for ink formulation. The detailed methods used for DBM powder preparation are provided in the Supporting Information (Supporting Methods and Figure S1). PCL pellets were dissolved in DCM under ultrasonic conditions at 50 °C for 30 min, followed by stirring and cooling to create the PCL ink (Figure S2A) suitable for printing. The DBM/PCL ink (Figure S2B) was produced with a two-step approach. First, plain PCL ink was prepared as previously mentioned, and then, DBM powder dispersed in minimal amounts of DCM was poured into the prepared PCL ink to make a final weight ratio of 70% DBM to 30% PCL. The composite ink was briefly placed in a room-temperature ultrasonic bath to create a homogeneous mixture. Finally, the DBM/PCL composite ink was subjected to constant stirring in an open-air environment (i.e., chemical fume hood) until the solutions reached an ideal viscosity (through solvent evaporation) for pneumatic printing (low shear stress viscosity of 30–35 Pa·s), as reported previously.^29^ For printing, the inks were transferred to separate 3 mL syringes equipped with a connective hose and a conical nozzle (G22, 410 μm). The syringes and nozzles used were purchased from CELLINK (Gothenburg, Sweden).

Circular, thin-film scaffolds were designed using AutoCAD (Autodesk, San Rafael, CA, USA). The 3D CAD model was created with a 14 mm diameter and a thickness of roughly 300 μm. The dimensions of the scaffolds were chosen to fit within a single well of a 24-well plate (15.6 mm diameter) for conducting in vitro testing while permitting easy removal for sample collection and biological analysis. The 3D CAD model was exported as a stereolithography (.stl) file and uploaded to a BIO X 3D pneumatic printer (CELLINK) for printing. Scaffolds were produced with a printing pressure of 200 kPa and a printing speed of 10 mm/s.

**Figure 1:**
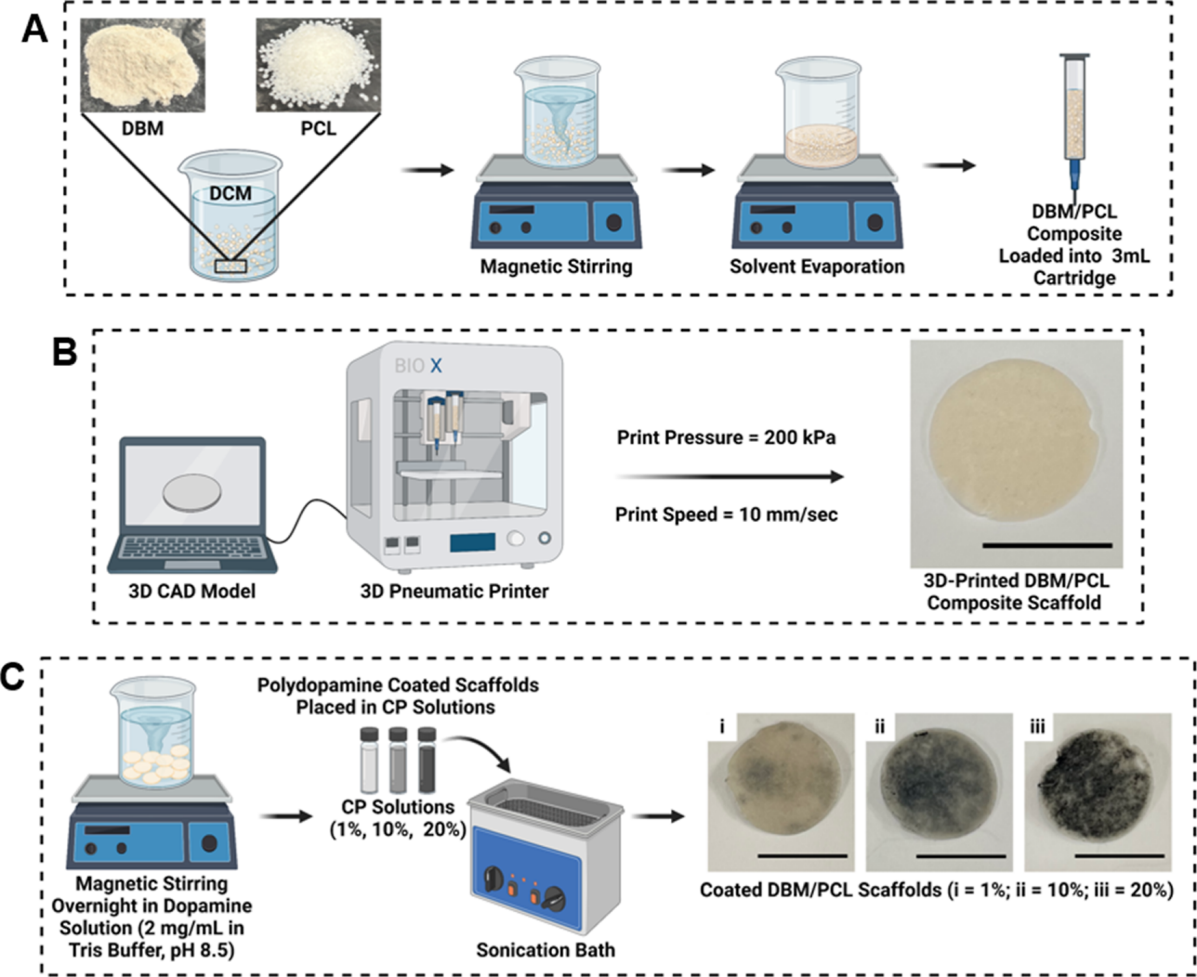
Overview of the (A) printing ink formulation, (B) composite scaffold fabrication utilizing a 3D pneumatic printer, and (C) conductive coating process, where 1, 10, and 20% are dilutions from a stock (4:1 weight ratio of PEDOT/PSS to PPy; 1.54 wt % at a 100% concentration).

To enhance scaffold conductivity, a combination of conductive polymers, PEDOT/PPS and PPy, was grafted onto the surface of the 3D-printed composite scaffolds using a PDA-mediated adhesion strategy. Briefly, scaffolds were first rinsed with DI water before being soaked overnight in a dopamine hydrochloride solution (2 mg/mL in 10 mM Tris buffer, pH 8.5) under constant stirring at 700 rpm at room temperature. After being immersed in the solution overnight, scaffolds were rinsed several times with DI water and left to dry. The PDA-modified DBM/PCL scaffolds were then coated with several dilutions (i.e., 1, 10, and 20%) of an optimized conductive polymer solution (PEDOT/PSS–PPy). The conductive polymer solution has a final weight ratio of 4:1 PEDOT/PSS to PPy and at 100% concentration is 1.54 wt % in DI water. Figure 1 provides an overview of the printing ink formulation, scaffold fabrication, and coating process used to create the conductive DBM/PCL composite scaffolds for this study.

### 2.3 Scaffold Characterization

To evaluate certain physical, biological, chemical, and electrical properties of the 3D-printed scaffolds, several techniques were utilized and detailed as follows.

#### 2.3.1 Tensile and Compressive Testing

The tensile and compressive properties of both the PCL and DBM/PCL composites were examined using a universal testing machine (ESM303, Mark-10, Copiague, NY, USA). For tensile properties, dog bone-shaped samples (*n* = 4) of both materials were produced with a gauge length of 12.7 mm, a gauge width of 6.35 mm, and a gauge thickness of 1 mm. Tensile tests were performed by stretching at a speed of 1 mm/min for the preloading and 5 mm/min for the loading conditions, respectively. After performing tensile tests, stress–strain curves were plotted and used to calculate the following: tensile modulus, ultimate tensile stress, ultimate tensile strain, and both the yield stress and yield strain using the modulus slope at a 0.2% strain offset. For compressive properties, cylindrical samples (*n* = 4) of both materials were created with a diameter of 5 mm and a height of 5 mm. Each specimen was compressed to a maximum strain of 60% at a crosshead velocity of 1 mm/min between two steel plates. After plotting the stress–strain curves, the compressive modulus was measured with a 0.2% strain offset linear slope technique and the compressive strength was calculated with respect to the compressive stress at 60% strain.

#### 2.3.2 Scanning Electron Microcopy

Scaffold surface morphology was studied using a field emission scanning electron microscope (FE-SEM; FEI Teneo, Thermo Fisher Scientific, Waltham, MA, USA). Thoroughly dried scaffolds were mounted onto stages using double-sided carbon tape before being coated with 20 nm of Au–Pd under vacuum via a Leica EM ACE600 sputter coater (Buffalo Grove, IL, USA). Surface imaging of the scaffolds was conducted at an acceleration voltage of 5 kV.

#### 2.3.3 Water Contact Angle

In addition to assisting with anchoring the conductive polymer coatings, PDA was deposited onto the surface of the 3D-printed scaffolds to encourage cellular adhesion. In order to assess scaffold wettability before and after the PDA coating, static water contact angle measurements were taken using a contact angle goniometer (Ossila, Sheffield, England, UK). Immediately after placing a single water droplet (10 μL) onto the surface of the scaffolds, photos were captured, and the resultant water contact angle (*n* = 3 per group) was measured using the instrument software.

#### 2.3.4 Fourier Transform Infrared Spectroscopy

Fourier transform infrared spectroscopy (FTIR) analysis was conducted using a Nicolet 6700 FT-IR spectrometer (Thermo Fisher Scientific) to characterize the functional groups and bonding between different scaffolding compositions and coatings (i.e., PCL, DBM/PCL, and PDA-coated DBM/PCL). The spectrometer was set to collect absorbance readings across the infrared spectra from 4000 to 700 cm^–1^. A total of 256 scans were collected for each prepared sample, and the spectral resolution was set to 6 cm^–1^.

#### 2.3.5 Scaffold Sheet Resistance

The electrical properties of DBM/PCL scaffolds coated with various concentrations of the optimized PEDOT/PSS–PPy coating were measured using a Four-Point Probe System (Ossila) at a voltage sweep of 1–10.50 V. Sheet resistance was calculated by placing the coated scaffolds onto glass slides and centering them on the testing stage beneath the probe head. A total of 10 readings were taken and averaged for each sample (*n* = 3 per group). The current was set to autorange with a current limit of 50 mA.

### 2.4 Cell Culture and Seeding

hMSCs between passage 4 and passage 6 were revived from cryopreserved stocks and expanded to experimental numbers before being used. Cells were grown in low-glucose DMEM supplemented with 10% FBS and 1% Pen–Strep, denoted as growth medium. Monolayers presenting roughly 90% subconfluency were passaged via treatment with trypsin–EDTA, formation of cell pellets by centrifugation (1200 rpm, 5 min), and resuspension in fresh growth medium. In an effort to minimize cellular attachment to the surface of culture wells and improve cellular adhesion potential to the scaffolding surfaces, Ultra-Low Attachment well plates were used. For cell seeding, sterilized (UV treated for 30 min per side) scaffolds were placed into wells and prewetted using 250 μL of growth medium. After counting, a cell suspension was created and used to seed wells for experiments. To bring the final culture volume in each well up to 500 μL, 250 μL of the cell suspension was added to all experimental and control wells. Culture plates were stored in a standard 5% CO_2_ incubator system at 37°C. The culture medium was changed every 3–4 days as recommended by the manufacturer.

### 2.5 Scaffold Biocompatibility Assessment and Cellular Proliferation Assay

#### 2.5.1 LIVE/DEAD Staining

The cytotoxicity of a range of conductive coating dilutions was analyzed after 2, 5, and 7 days of culture in growth medium. Cells were seeded at a density of 5 × 10^3^ cells/cm^2^ on scaffolds placed in 24-well plates and stored inside an incubator. At specified time points, a qualitative assessment of cell viability was performed using a LIVE/DEAD Viability/Cytotoxicity Kit (Molecular Probes, Invitrogen, Eugene, OR, USA) according to the specifications of the manufacturer. Briefly, the culture medium was removed from individual wells and the cell-laden scaffolds were washed with sterile PBS. After completely covering each scaffold with a working dilution of calcein AM and ethidium homodimer-1, cells were incubated at room temperature for 30 min, protected from light. Before imaging, the working reagent was replaced with sterile PBS and the scaffolds were inverted. Images were taken with an EVOS FLc Cell Imaging System (EVOS, Thermo Fisher Scientific) with the magnification set to 10× for all images. Live cells were captured under a GFP filter, and dead cells were detected by viewing them with a Texas Red filter.

#### 2.5.2 CCK-8 Assay

Proliferation was measured using a Cell Counting Kit-8 (CCK-8, Sigma-Aldrich) colorimetric assay to determine whether a range of conductive coating dilutions had any adverse effects on cellular growth. Cells were seeded at a density of 5 × 10^3^ cells/cm^2^ on scaffolds placed in 24-well plates and stored inside an incubator. For the assay, a standard curve of known viable cells ranging from 0 to 100,000 cells was first created as instructed by the manufacturer. At individual time points, the CCK-8 solution was added into the scaffold containing wells at a volume equivalent to 10% of the culture medium (i.e., 50 μL). The plates were incubated for 1 h at 37°C. The absorbance of the samples and standards was measured at 450 nm using a Cytation 1 imaging reader (BioTek, Agilent Technologies, Santa Clara, CA, USA).

### 2.6 ES Chamber and Sterilization

For this study, stimulation was applied via a purpose-built 24-well DC ES cell culture chamber using methods established previously.^30^ The ES culture chamber consists of two 34-mm long L-shaped platinum electrodes per well placed precisely 10 mm apart. The bottom end of each electrode is 10 mm in length; the 24 mm top end of each electrode extends through the culture plate lid and is fixed in place using silicone glue. The electrodes are soldered into a parallel circuit using silver wire and connected to a tunable DC power source via alligator clips. Prior to each round of ES treatment, the electrodes were soaked in 70% ethanol for 15 min before being washed with sterile PBS. The lid with fixed electrodes was then inverted and exposed to UV light in a laminar flow cell culture hood for 1 h.

### 2.7 Evaluation of Osteogenic Potential with ES

For osteogenic differentiation, hMSCs were cultured in high-glucose DMEM supplemented with 10% FBS, 1% Pen–Strep, 50 μg/mL ascorbic acid, 10^–7^ M dexamethasone, and 10 mM β-GP, denoted as differentiation medium. Cells were seeded at a density of 8 × 10^4^ cells/cm^2^ on scaffolds placed in 24-well plates and allowed to attach inside an incubator. After an overnight attachment period, the growth medium used for seeding was replaced with differentiation medium, and ES treatment began. Stimulation consisted of 100 mV/mm DC ES applied to seeded cells continuously for 5 min daily on alternating days (i.e., four times/week). In addition to evaluating osteogenic differentiation through mineral staining and protein analysis, cell viability was monitored throughout the 2 week osteogenic culture using both qualitative and quantitative methods, as outlined below.

#### 2.7.1 Cell Viability

To confirm that the electrochemical reactions and oxidation–reduction of the platinum electrodes were not cytotoxic, a qualitative and quantitative evaluation of cell viability was performed using a Hoechst 33342 (Molecular Probes, Invitrogen) stain and CCK-8 assay, respectively. After performing both assays according to manufacturer specifications, the stained cells were viewed under a DAPI filter at 4× magnification, and the sample absorbance was measured at 450 nm using a Cytation 1 imaging reader.

#### 2.7.2 Alkaline Phosphate Activity

An alkaline phosphatase (ALP) Assay Kit (abcam, Waltham, MA, USA) was the test used for the presence of osteoblast-like cells over the course of the 14 day long culture period. This assay employs *p*-nitrophenyl phosphate (pNPP) hydrolyzed by ALP to induce a color change (yellow-colored) related to ALP levels within the samples. Protein samples were collected by first removing the culture medium from test wells, rinsing with PBS, and dissociating cells from scaffolds by trypsinization. After confirming disassociation by visual inspection under a microscope, the reaction was neutralized with fresh culture medium, and the contents of each well were transferred to labeled microtubes. Cell suspensions in each microtube were centrifuged at 14,000×*g* for 15 min before removing the supernatant and resuspending in Mammalian Protein Extraction Reagent (M-PER, Thermo Fisher Scientific) to extract proteins from the suspended cells. Microtubes were once again spun down, and the supernatant was used for further analysis.

For the assay, a volume of 20 μL of each sample or 120 μL of each standard were pipetted into individual wells in a 96-well plate. A volume of 60 μL of the assay buffer was added to sample wells to bring the total volume up to 80 μL. Subsequently, 50 μL of prepared 5 mM pNPP solution was added to sample wells. The conversion of ALP within the standards was activated with 10 μL of the reconstituted ALP enzyme, and the plate was incubated at room temperature in the dark for exactly 1 h. The conversion was stopped with the addition of 20 μL of the stop solution to each well. The absorbance was measured at 405 nm using a Cytation 1 imaging reader.

#### 2.7.3 Osteocalcin ELISA Assay

A Human Osteocalcin ELISA Kit (#EKU06413, Biomatik, Wilmington, DE, USA) was used to measure the expression of osteocalcin, a protein indicative of osteoblast maturation, as directed by the manufacturer’s protocol. Protein samples were prepared as described in the previous section. For the assay, 100 μL of the prepared standards and experimental protein samples were pipetted into a precoated 96-well strip plate and placed into an incubator at 37°C for 1 h. Next, the protein samples and standards were replaced with 100 μL of reagent A, and the plate was placed back in the incubator for 1 additional hour at 37°C. Following the second incubation, the plate was washed with a diluted wash buffer, reagent B was added, and, then, the plate was incubated for 30 more minutes at 37°C. Then, 90 μL of substrate solution was added, and the plate was incubated for a final time (20 min at 37°C). Lastly, 50 μL of stop solution was added to all wells to produce a final color change before using a Cytation 1 imaging reader to read the absorbance at 450 nm.

#### 2.7.4 Xylenol Orange Mineral Staining

To stain mineralized nodules within the different groups, xylenol orange (XO), a nondestructive calcium-chelating fluorescent stain, was used as previously described with minor deviation.^31^ A 20 mM stock solution was created using DI water and added to specific wells at a volume equivalent to 1% of the culture medium and allowed to incubate overnight. Prior to imaging, the culture medium was replaced with sterile PBS and the scaffolds were inverted. Calcium deposition on scaffolds was detected using a Cytation 1 imaging reader equipped with a TRITC filter. The objective magnification on the microscope was set to 4× for all images.

### 2.8 Statistical Analysis

All quantitative data reported was obtained using, at minimum, triplicates (*n* ≥ 3). Quantitative results are expressed as the mean with standard deviation (SD) indicated by error bars. The statistical analysis was completed using GraphPad Prism (GraphPad Software, Inc., San Diego, CA, USA). Two-way ANOVA was performed followed by Tukey post-tests for multiple comparisons to determine statistical differences between individual sample groups at various time points; *p* < 0.05 was considered to be statistically significant.

## 3 Results and Discussion

Hard tissue repair and regeneration is a persistent clinical challenge, costing hundreds of billions of dollars annually, and with an aging population, the surgical need to repair these tissues has increased considerably.^32−34^ Therefore, significant efforts have been made in tissue engineering and regenerative medicine to meet this urgent need, notably the use of “smart” materials as scaffolding for bone. These biomaterials mimic the physiochemical properties of natural bone tissue, having instructive or stimulating effects on cells/tissues, and can be designed with individually tailored properties to actively participate in tissue regeneration.^35,36^ The results from this work suggest the promising potential of combining human allograft tissues with conductive polymers for the development of biomimetic scaffolding constructs capable of: (1) replicating both the biological and electrical properties of healthy bone tissue and (2) facilitating the transfer of exogenous electrical signals to growing cells to elicit an improved osteogenic response over nonconductive DBM-loaded scaffolds in vitro.

### 3.1 Scaffold Characterization

#### 3.1.1 Tensile and Compressive Properties of PCL and DBM/PCL Composites

PCL is an FDA-approved linear polyester with decent biocompatibility, improved degradation characteristics compared to those of similar polymers, and load-bearing capabilities, contributing to its frequent use as a material for synthetic bone.^37,38^ Although PCL presents mechanical properties favorable for producing bone scaffolding, PCL has no bioactivity, thus rendering it incapable of inducing bone regeneration alone.^39,40^ Therefore, numerous studies combining metals, oxides, polymers, and carbon-based materials with PCL for property improvement have been conducted with their achievements reported elsewhere.^41^ In this study, we evaluate the effectiveness of combining DBM and a blend of conductive polymers with PCL to improve scaffolding characteristics related to bone tissue engineering applications.

**Figure 2:**
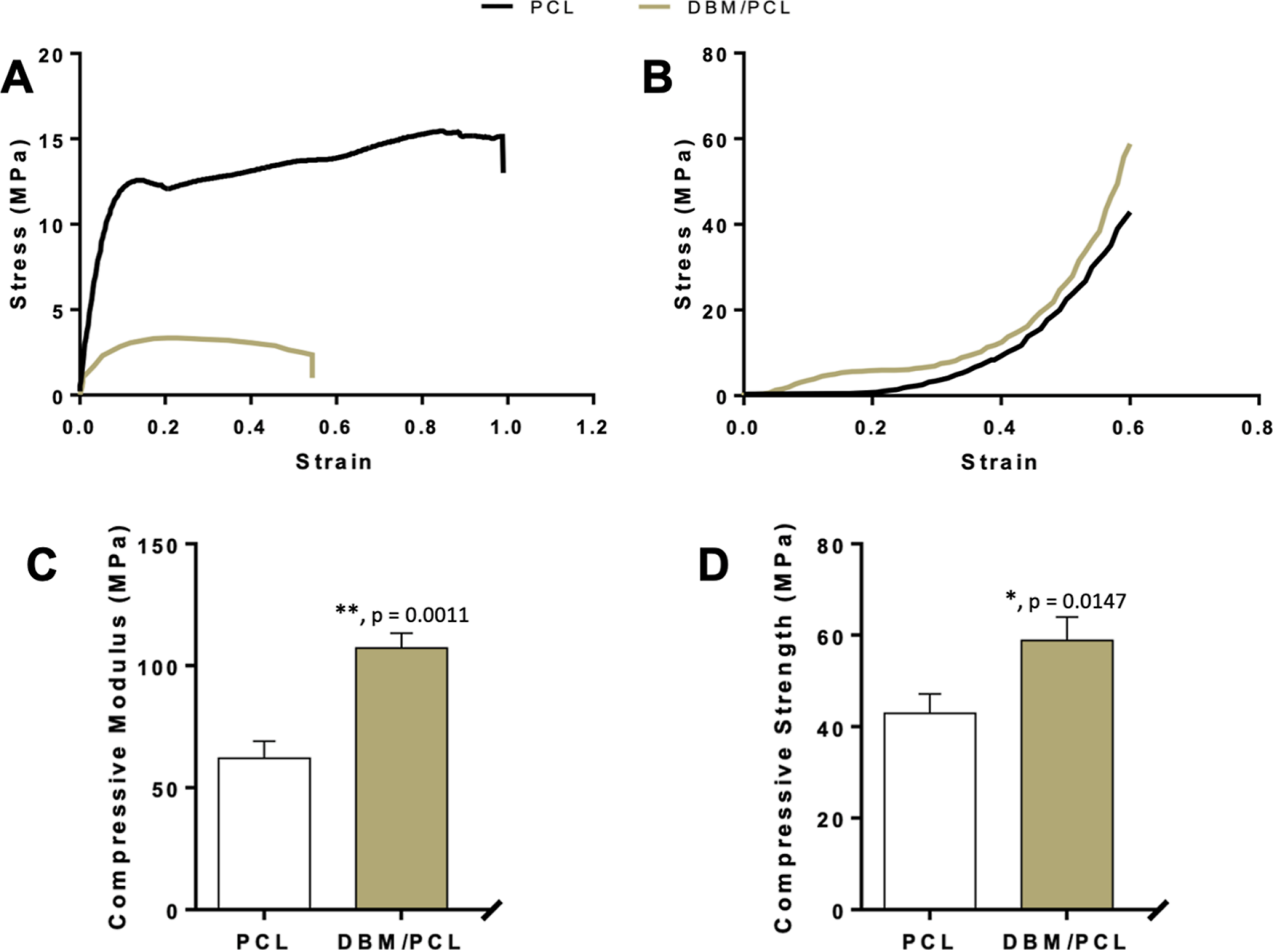
Mechanical properties of PCL and DBM/PCL composites. (A) Tensile and (B) compressive stress–strain curves. Comparison of the (C) compressive modulus and (D) compressive strength of PCL and DBM/PCL composites estimated at 60% strain.

To explore the effects that DBM had on the mechanical properties of the scaffolding materials, both tensile and compressive tests were performed. The tensile stress–strain curves for PCL and DBM/PCL composites are shown in Figure 2A. PCL showed typical amorphous polymer behavior with a prolonged strain-hardening phase, indicating that PCL is a ductile polymer that possesses good toughness. After incorporating the prepared DBM powder (<125 μm) into the scaffolding matrix (70% by weight), a significant change was observed in the tensile properties, as reported in Table 1. The tensile modulus of the DBM/PCL composite was found to be 38.547 ± 1.401 MPa, whereas that of PCL was 163.54 ± 4.717 MPa. Similarly, the ultimate tensile strength of the composite decreased to 3.33 ± 0.176 MPa from 15.465 ± 0.669 MPa for the pure PCL. Various studies have investigated the changes in the tensile characteristics of PCL with the addition of nano- and/or microparticles, concluding that in excessive amounts, particle agglomeration creates defects in the material’s surface and, eventually, results in a reduction of tensile strength.^42−44^ Interestingly, studies conducted by Goldstein et al.,^45^ Behrens et al.,^46^ and Lindahl^47^ attempting to characterize the tensile properties of human bones have reported results with wide variation, finding that bone tissue has tensile strength ranging from 0.2 to 63.6 MPa. Thus, based on tensile testing, our composites provide tensile properties suitable for applications related to bone tissue engineering.

**Table 1:** Tensile Properties of PCL and DBM/PCL Composites^a^

	Composition	
Property	PCL	DBM/PCL
Tensile modulus (MPa)	163.54 ± 4.717	38.547 ± 1.401
Yield stress (MPa)	12.5 ± 0.826	2.312 ± 0.092
Yield strain	0.127 ± 0.02	0.053 ± 0.007
Ultimate tensile stress (MPa)	15.465 ± 0.669	3.33 ± 0.176
Ultimate tensile strain	1.058 ± 0.714	0.59 ± 0.015

a Values are represented as mean ± SD (*n* = 4).

Figure 2B shows the stress–strain curves obtained from uniaxial compressive testing of PCL and DBM/PCL composites. The calculated compressive modulus and estimated compressive strength for PCL and DBM/PCL are shown in Figure 2C,D, respectively. The compressive modulus for DBM/PCL is 107.2 ± 6.1 MPa, while the compressive modulus for PCL is just 62.1 ± 6.3 MPa. In addition to an increase in the compressive modulus, the incorporation of DBM significantly improved the compressive strength of DBM/PCL to 58.82 ± 5.17 from 42.93 ± 4.25 for plain PCL. Compared with PCL, the compressive modulus and compressive strength of the DBM/PCL composites increased by approximately 1.73 and 1.37 times, respectively. The compressive modulus of cancellous bone is reported to range from 10 to 2000 MPa;^48^ thus, the improved compressive properties of the DBM/PCL composite suggest its usefulness for producing scaffolds intended for bone regeneration.

#### 3.1.2 Surface Morphology

PCL is relatively hydrophobic in nature and is usually modified prior to being utilized as scaffolding for biological applications. For PCL scaffolds, surface roughness is an easily tailorable and effective factor for influencing cellular behavior; thus, the surface morphology of scaffolds loaded with DBM were analyzed via SEM (Figure S3). Representative SEM images of the unmodified DBM/PCL scaffolds and those coated with various dilutions (i.e., 20, 10, and 1%) of the conductive coating depict a qualitative assessment of the influences that DBM and the conductive coatings had on the surface properties (i.e., roughness) of the experimental scaffolds. Unsurprisingly, the surface homogeneity of the scaffolds was altered with the incorporation of DBM into the scaffolding matrix. Uncoated DBM/PCL scaffolds exhibited a textured surface that was almost uniformly covered by micro/nanopores (Figure S3B). This was a welcomed finding as rough surfaces encourage the entrapment of various proteins and can contribute to increased adhesion and proliferation of osteogenic cells.^49^ With the addition of the conductive coating, even at a very dilute amount (i.e., 1%, Figure S3C), the surface morphology of the scaffolds appeared smooth, reminiscent of pristine PCL scaffolds (Figure S3A). From this SEM analysis, we were able to conclude that the incorporation of DBM significantly altered the surface roughness of our scaffolds; however, with the addition of a uniform conductive polymer coating, the surface topography was similar to that of unmodified PCL scaffolds.

#### 3.1.3 Scaffold Wettability and Chemical Characterization

**Figure 3:**
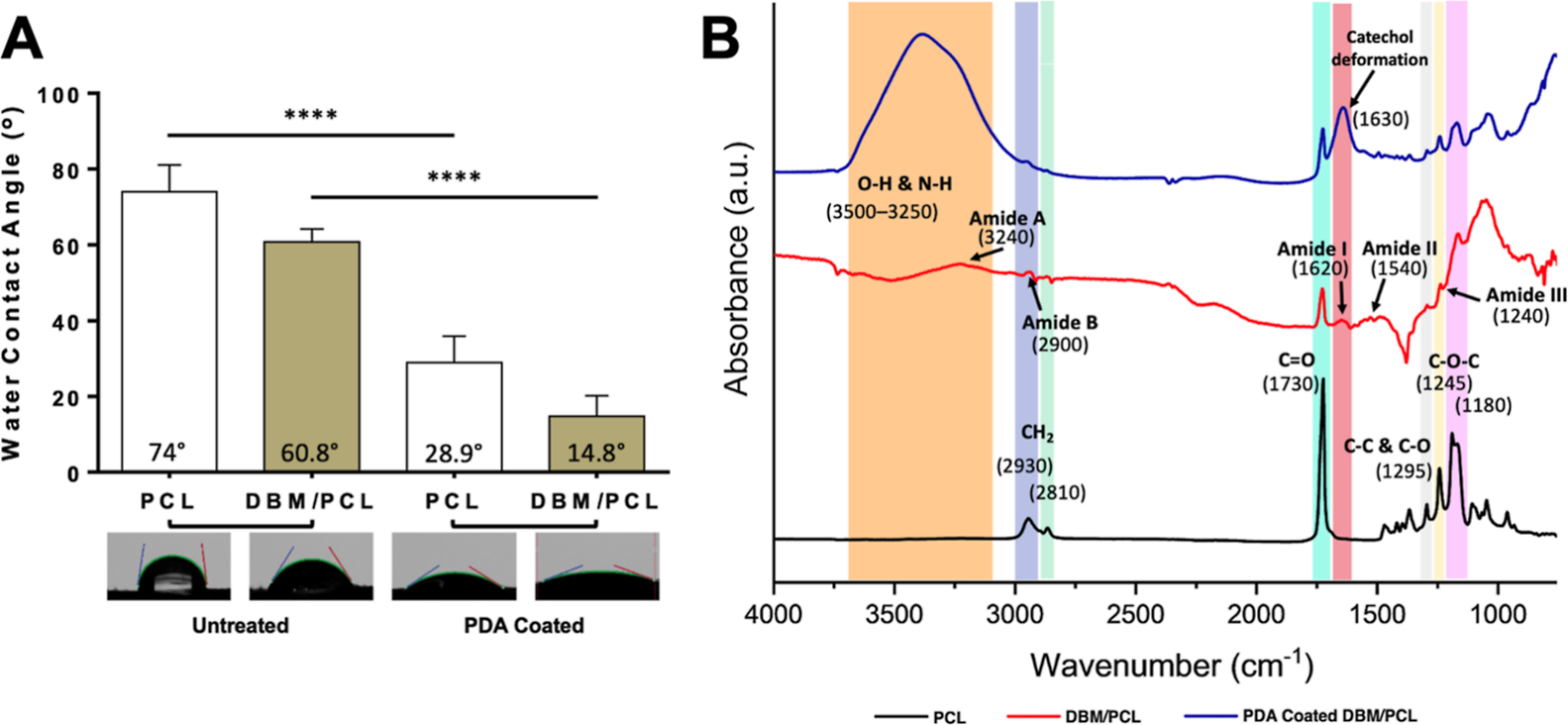
Surface properties of 3D-printed scaffolds. (A) Scaffold wettability as determined through static water contact angle measurements. (B) FTIR spectra of PCL, DBM/PCL, and PDA-coated DBM/PCL scaffolds.

The wettability of a material is a determining factor for protein absorption and cellular adhesion; consequently, surfaces with moderate hydrophilicity usually improve cell growth/material biocompatibility compared to those with exceedingly hydrophilic (θ < 5°) or hydrophobic (θ > 150°) surfaces.^50^ In addition to assisting with anchoring the conductive polymer coatings, mussel-inspired PDA was deposited onto the surface of the 3D-printed scaffolds to encourage cellular adhesion. PDA coatings have been shown to improve factors related to the osteogenic differentiation of various cell types used in scaffold-based bone tissue engineering approaches and therefore applied to this work.^51−53^ Water contact angle analyses demonstrating the wettability of the 3D-printed scaffolds before and after the PDA coating are shown in Figure 3A. Uncoated PCL and DBM/PCL scaffolds exhibited water contact angle measurements of 74 ± 7.15 and 60.8 ± 3.45°, respectively. After the PDA coating, a significant decrease in the water contact angle measurements for each scaffold type (PCL, 28.9 ± 7°, and DBM/PCL, 14.8 ± 5.4°) was observed, indicating greater surface wettability and, subsequently, improved cellular affinity to the 3D-printed scaffolds.

FTIR spectra of PCL, DBM/PCL, and PDA-modified DBM/PCL scaffolds are shown in Figure 3B. This analysis was conducted to identify the functional groups present on the surface of the different scaffolds and as a secondary verification (along with water contact angle measurements) that PDA had been deposited onto the scaffolds. The PCL spectra displayed asymmetric CH_2_ stretching vibration and symmetric CH_2_ stretching at 2930 and 2810 cm^–1^, respectively, and carbonyl (C=O) stretching vibration around 1730 cm^–1^. An additional band distinguishable from PCL related to C–C and C–O stretching vibration was identified at 1295 cm^–1^. The DBM/PCL spectra show the preservation of several PCL-associated peaks, such as that near 1730 cm^–1^ related to C=O stretching vibration. Also, new bands corresponding to the collagen Type I membrane amides^54^ were now identifiable, further confirming the demineralization of the bone tissue samples within the scaffolding matrix. Following the PDA coating, changes were observed in the spectra for DBM/PCL and PCL (Figure S4) scaffolds. Notably, the broad peak between 3250 and 3500 cm^–1^, indicating O–H and N–H bonds in the catechol groups, as well as a new peak around 1630 cm^–1^ contributed to PDA catechol deformation.

#### 3.1.4 Scaffold Electrical Properties

Conductive bone scaffolds can transfer electrical and electrochemical signals to targeted cells, providing clinical advantages over nonconductive polymer-based scaffolds and those loaded with highly soluble growth factors.^55^ In this work, we utilize a PDA-mediated coating strategy to anchor a blended PEDOT/PSS–PPy solution onto 3D-printed DBM/PCL composite scaffolds to enhance their conductive properties and osteogenic performance. Previous studies have utilized 3D printing and other additive manufacturing techniques to prepare DBM-infused scaffolds, assessing their mechanical properties and regenerative potential both in vitro and in vivo.^56−59^ To our knowledge, however, no studies have developed DBM-infused scaffolds possessing measurable electrical conductivity for bone regeneration. Given this, we aimed to develop a biomimetic hybrid scaffold with enhanced bioactivity and electrical conductivity.

**Figure 4:**
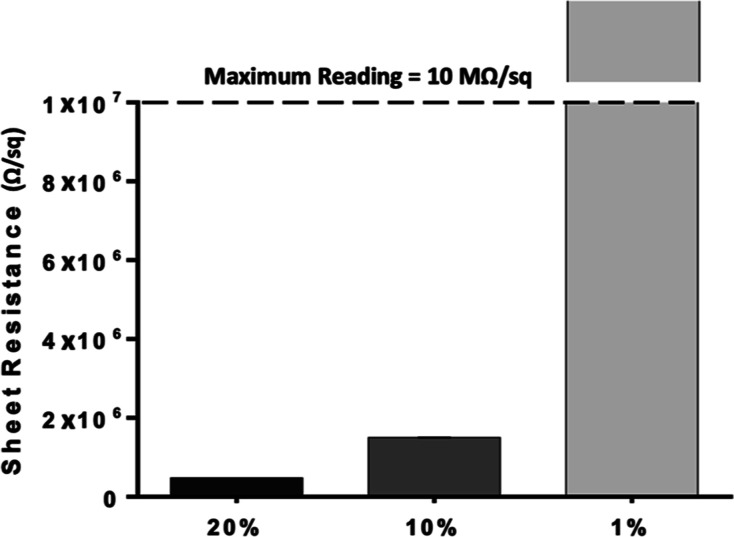
Sheet resistance measurements of DBM/PCL scaffolds coated with different dilutions of a PEDOT/PSS–PPy conductive polymer solution (1.54 wt % in DI water at 100% concentration) with a 4:1 weight ratio.

Our experimental conductive polymer solution has a final weight ratio of 4:1 PEDOT/PSS to PPy and at 100% concentration is 1.54 wt % in DI water. Being two of the most studied conductive polymers, PPy was chosen for its outstanding electrical and stimuli-responsive properties, while PEDOT was selected due to its high chemical and electrical stability.^24^ The electrical properties of DBM/PCL scaffolds coated with different concentrations of the optimized conductive coating were studied by measuring the sheet resistance using a four-point probe method. Scaffolds formed of pure PCL using various techniques have been reported to have negligible conductivity, and thus, the electrical properties of the uncoated scaffolds used in this study were not characterized.^60,61^ Sheet resistance is inversely proportional to conductivity; therefore, measurements reported in Figure 4 conclude that scaffold conductivity is enhanced with increased coating concentrations. The 1% coated DBM/PCL scaffolds were found to be minimally conductive, if at all, with an average sheet resistance exceeding the equipment measurement range (>10 MΩ/sq). The average sheet resistance for the 10% and 20% coated scaffolds was found to be 1.5 × 10^6^ and 4.77 × 10^5^ Ω/sq, respectively. Although the electrical properties of the scaffolds could be further improved by using a more concentrated coating dilution, it should be noted that excessive amounts of conductive materials can become toxic to living cells.^19^

### 3.2 Cell Viability and Proliferation on Conductive Scaffolds

**Figure 5:**
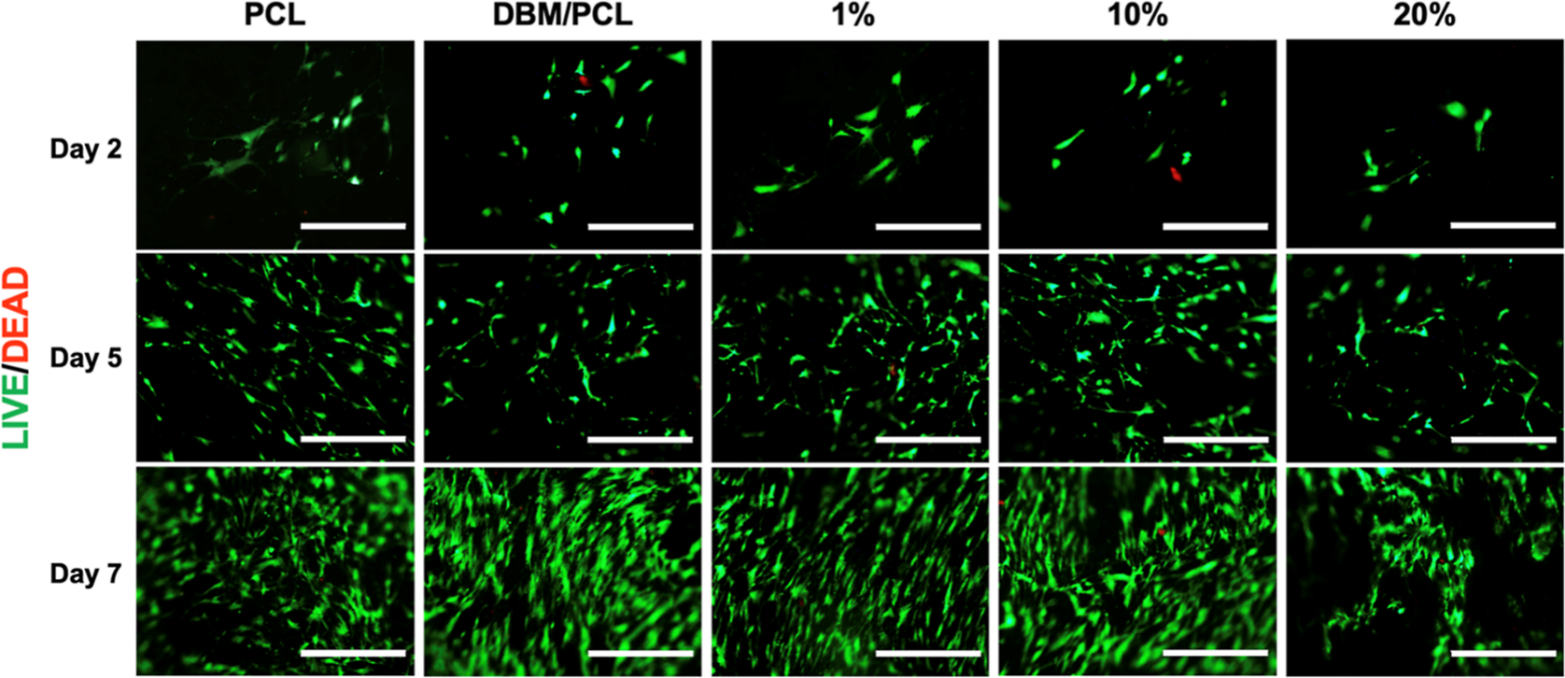
LIVE/DEAD staining shows qualitative cell viability and proliferation of hMSCs grown on PCL, DBM/PCL, and conductive DBM/PCL scaffolds over 7 days. Staining confirms that cells grown on DBM/PCL scaffolds with various conductive coatings (i.e., 1, 10, and 20%) remained viable throughout the entire culture period. Here, the conductive coatings represent dilutions from a stock (4:1 weight ratio of PEDOT/PSS to PPy; at 100% concentration, the coating is 1.54 wt % in DI water). Scalebars = 400 μm.

**Figure 6:**
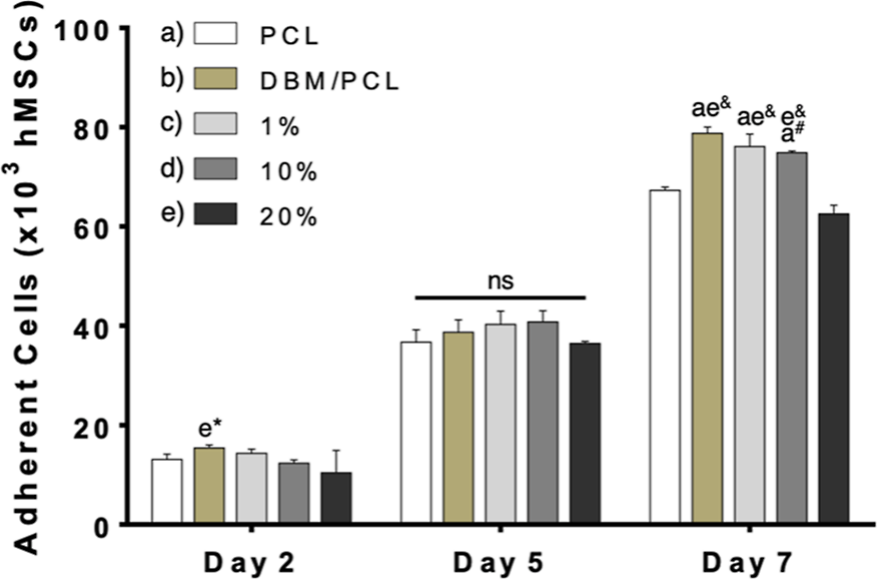
Adherent cells on PCL, DBM/PCL, and conductive DBM/PCL scaffolds after 2, 5, and 7 days of culture. The conductive coatings (i.e., 1, 10, and 20%) represent dilutions from a stock (4:1 weight ratio of PEDOT/PSS to PPy; 1.54 wt % in DI water at 100% concentration). Differences between groups are represented by their corresponding letters and the level of significance is represented by symbols (*p < 0.05; #*p* < 0.001; &*p* < 0.0001).

The viability and proliferation of hMSCs grown on composite scaffolds coated with various conductive coating dilutions were investigated to determine an optimum concentration for experiments utilizing ES. LIVE/DEAD staining, shown in Figure 5, indicated high cell viability in all scaffolding groups, with very few dead (red-stained) cells present. Further, staining results concluded that scaffolds coated with dilutions up to 20% were able to support cell attachment and proliferation, as demonstrated by the increased presence of live (green-stained) cells from Day 2 to Day 5 and from Day 5 to Day 7 on all scaffolds. To quantify the growth of cells over time, the number of adherent cells was determined using a CCK-8 assay. Results from the cell counting assay (Figure 6) support qualitative findings; there was an increase in the cell number at each successive time point for all scaffolding groups, as is expected for actively proliferating cells. Interestingly, uncoated DBM/PCL scaffolds had significantly more (*p* = 0.0325) adherent cells present on Day 2 when compared to those coated with the 20% conductive solution. By Day 7, uncoated composite scaffolds and those coated in low concentrations (1 and 10%) had significantly more adherent cells on their surfaces when compared to PCL control scaffolds and composite scaffolds coated with the 20% conductive solution. These findings could be attributed to the enhanced biological and surface properties of scaffolds with DBM and the potentially excessive amounts of conductive polymers in the 20% coated DBM/PCL scaffold group. Yang et al.^62^ report that DBM particle size can influence the proliferation of osteoblastic cells, noting that smaller-sized particles (in the micron range) may provide surface roughness beneficial to cellular growth. Therefore, the DBM powder (<125 μm) used in this study may afford a similar benefit. With respect to the 20% coated scaffolding group, Wibowo et al.^63^ describe results related to the attachment and proliferation of human-derived cells grown on scaffolds with different electrical properties comparable to our findings. Due to these observations and the electrical properties reported earlier (Section 3.1.4 and Figure 4), the diluted coating of 10% concentration was chosen for experiments where cells were exposed to ES.

#### 3.2.1 In Vitro Analysis of Osteogenic Differentiation with ES

The osteoinductivity of scaffolds designed for bone tissue regeneration is an essential attribute. Recent studies in our group detail the promising future that conductive materials present toward alleviating issues related to bone repair and regeneration and how, in conjunction with ES, scaffolds with improved electrical properties can enhance in vitro mineralization.^30,55^ Here, the ability of our electrically conductive DBM/PCL composite scaffolds to promote osteogenic differentiation of hMSCs when combined with ES was evaluated. Cells were cultured with or without stimulation to observe the influence of the conductive composite scaffolds and ES on the expression of both early- (7 days) and late-stage (14 days) osteogenic markers and mineralization.

**Figure 7:**
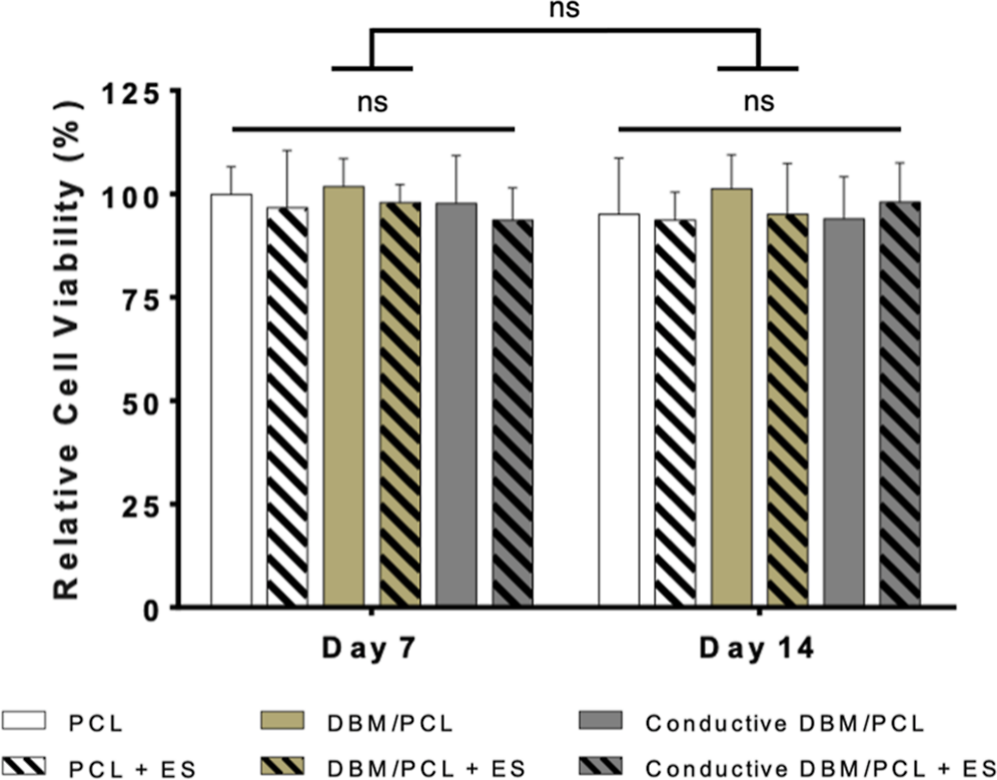
Relative cell viability over the 14 day osteogenic culture. No significant differences were observed, indicating that the ES provided (100 mV/mm for 5 min/day four times/week) was not detrimental to the viability of the seeded cells. Reported data is normalized to the unstimulated PCL group at Day 7.

In most in vitro applications, cells are subjected to ES provided by tailor-made platforms designed to fit the specific needs of different research groups.^64^ Similarly, for this work, stimulation was applied via a purpose-built 24-well DC ES cell culture chamber with previously optimized parameters (100 mV/mm for 5 min/day four times/week). Though the methods used in this study were already validated, cell viability was assessed throughout the culture to confirm that the electrochemical reactions and oxidation–reduction of the platinum electrodes in our platform were not cytotoxic. Figure 7 shows the viability assay results, which concluded that with or without ES, cells seeded on experimental (DBM/PCL and conductive DBM/PCL) scaffolds remained as viable as those grown on unstimulated PCL control scaffolds. These findings provide additional confirmation that the chosen field strength of 100 mV/mm, which McCaig et al.^65^ report to be sufficient for stimulating cells, is not detrimental to the viability of hMSCs grown on our composite scaffolds. Furthermore, several studies using similar levels of stimulation involving various cell types (derived from rats and humans), conducted both in monolayer and on conductive scaffolding surfaces, report sustained or even improved viability over that of unstimulated cells.^66−68^ Interestingly, in the study conducted by Shi et al.,^68^ they found that compared to that of unstimulated groups, the viability of human dermal fibroblasts grown on conductive membranes could be increased between 2.2 and 4 times simply by extending the time (up to 24 h in this study) of ES (100 mV/mm) exposure. These observations suggest that stimulation duration, particularly at relatively low field strengths (i.e., 100 mV/mm), in conjunction with conductive scaffolding, could have modulatory effects on cell viability. Our previous results^30^ and those reported in the current study conclude that short-term stimulation (∼5 min/day) in conjunction with conductive scaffolding has no significant effect on cell viability but is sufficient for improving the osteogenic response of bone-derived mammalian cells. In published work by Liu et al.,^69^ they investigate the effects of ES voltage/duration on different cellular processes (e.g., proliferation and osteogenic differentiation) of murine preosteoblasts (MC3T3-E1 cells) grown on PPy electrodes. The data they report suggests that a low stimulation voltage/duration (15 mV for 30 min/day) may be beneficial for cell growth, but the differentiation of these cells favors a slightly more prolonged exposure (∼1 h/day). Hence, further studies need to be conducted to better understand how various ES regimens induce changes in different cellular functions, particularly those useful in bone regeneration.

DBM consists of a collagen Type I network containing essential bone-related growth factors, such as BMPs, which remain after removing the cellular and mineral components of bone tissue.^21,22^ As an alternative to incorporating freely released growth factors, we present a simple and effective method for enhancing the biological properties of synthetic scaffolding constructs using DBMpowder (<125 μm) originating from human allograft tissues. It has been shown that DBM particle size plays an important role in osteoblastic cell functions; Yang et al.^62^ note clear correlations between osteogenic differentiation of MC3T3-E1 cells and the size of the DBM particles they were cultured in the presence of. Their study found that particles in the micron range (<1000 μm) provided the most favorable platform for osteogenic differentiation. This intriguing observation and those mentioned previously regarding the effects of conductive biomaterials and ES on various cell functions provide a significant foundation for our scaffold design. As shown, ES, conductive biomaterials, and DBM all separately influence aspects related to bone regeneration; thus, the combination of this triad could provide considerable benefit to bone tissue engineering approaches (i.e., biomimetic scaffold development). Thus, in conjunction with ES, the ability of the conducive DBM-infused scaffolds to induce osteogenic differentiation of hMSCs was evaluated.

**Figure 8:**
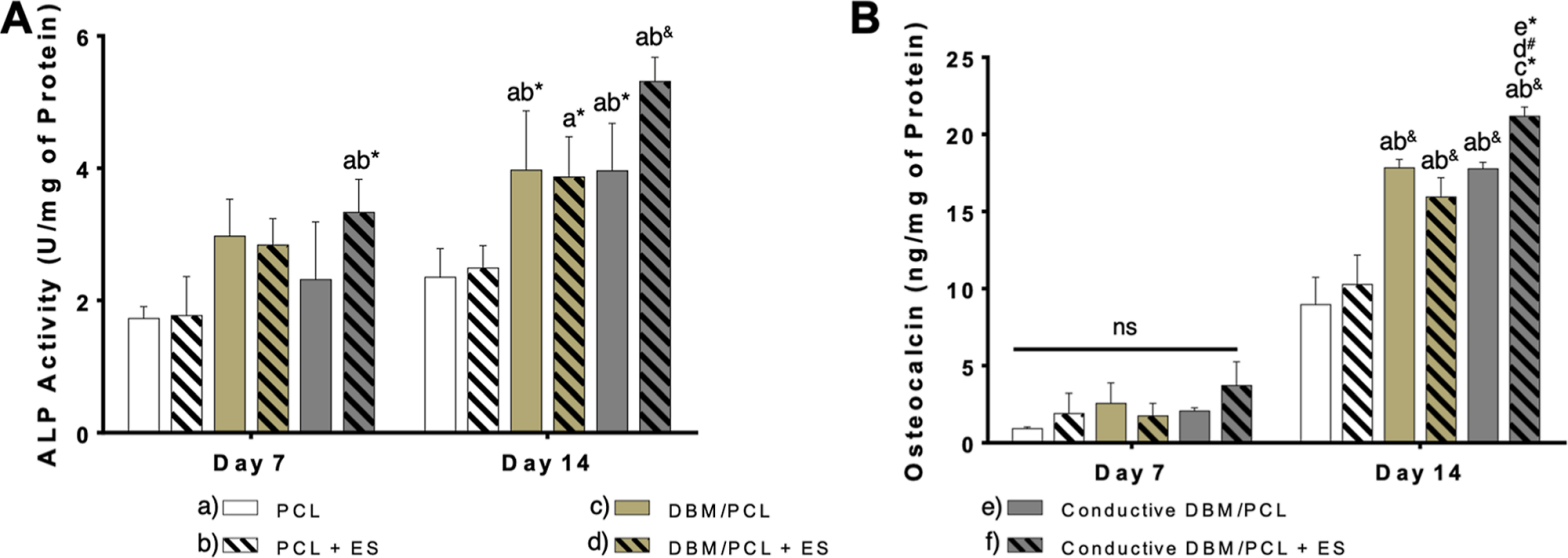
Early- and late-stage osteogenic marker expression over the 14 day osteogenic culture. (A) ALP activity of cells and (B) osteocalcin levels within each group with (100 mV/mm for 5 min/day four times/week) and without ES. Differences between groups represented by their corresponding letters and significance levels represented by symbols (*p < 0.05; #*p* < 0.001; & *p* < 0.0001).

ALP is one of the earliest markers of osteoblastic cell differentiation. Osteocalcin, the most abundant noncollagenous bone matrix protein, is often used as a late marker for bone formation.^70^ Therefore, ALP activity and osteocalcin levels within different scaffolding groups (both exposed to ES and not exposed to ES) were determined after 7 and 14 days of osteogenic culture to systematically assess the usefulness of the distinctive scaffolding components and methods used. Figure 8A shows the ALP activity corresponding to the different culture groups at Day 7 and Day 14. Although slight increases in ALP activity were noticed for all culture groups with DBM-containing scaffolds at Day 7, only conductive composite scaffolds exposed to ES displayed significance relative to PCL-only scaffolds. The combination of ES with conductive DBM/PCL composite scaffolds significantly enhanced ALP activity at Day 7 compared to that of unstimulated PCL control scaffolds (*p* = 0.0248) and PCL scaffolds that received ES (*p* = 0.0303), which may suggest that these components (i.e., ES, conductive materials, and DBM) together elicit this response but not separately or in pairs. By Day 14, ALP activity was significantly higher in all scaffolding groups with DBM compared to that in unstimulated PCL control scaffolds, with the conductive DBM/PCL scaffolding group that received ES showing the greatest level of significance (i.e., *p* < 0.0001) compared to unstimulated PCL control scaffolds. These results demonstrate the positive effects of combining ES, conductive materials, and DBM on early osteogenic differentiation.

The osteocalcin levels within the different culture groups at Day 7 and Day 14 are shown in Figure 8B. Mature osteoblasts preferentially express this matrix protein; thus, insignificant levels were expected after only 7 days of osteogenic culture regardless of the culturing conditions. However, by Day 14, osteocalcin levels were significantly higher (*p* < 0.0001) in all scaffolding groups with DBM compared to those in the unstimulated PCL control scaffolds and PCL scaffolds that received ES. This observation gives insights into the possible benefits that DBM has for improving the bioactive properties of synthetic scaffolding matrices, particularly those intended for bone regeneration. Of note, cells grown on conductive DBM/PCL composite scaffolds exposed to ES expressed significantly higher (at varying amounts) levels of osteocalcin compared to those of nonconductive DBM-infused scaffolds and conductive DBM/PCL scaffolds not receiving ES. Along with our previous report^55^ discussing the potential regenerative benefits of combining ES with conductive scaffolding and our subsequent study^30^ demonstrating the positive influence that this combination has on osteocalcin expression in vitro, these results provide further evidence that these components (i.e., ES, conductive materials, and DBM) all play some role in provoking a regenerative response in bone cells. However, further testing (e.g., using extended culture periods) should be conducted to gain more valuable insights into the effectiveness of this scaffolding approach toward clinical bone tissue engineering applications.

**Figure 9:**
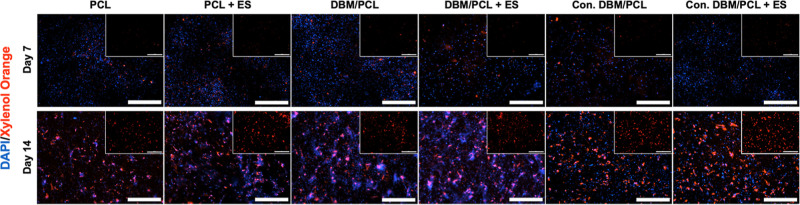
XO/DAPI staining with (100 mV/mm for 5 min/day four times/week) and without ES on PCL, DBM/PCL, and conductive DBM/PCL scaffolds after 7 and 14 days of osteogenic differentiation (the insert is XO alone). Scalebars = 500 μm.

Finally, mineralization (i.e., calcium deposition on scaffolds) was determined via XO staining. Figure 9 shows mineralized nodules within all groups marked distinctively in red, particularly by Day 14. Live cell nuclei are shown by DAPI overlay (marked in blue) to demonstrate qualitative cell viability throughout the culture and significant cell spreading on all surfaces (enhanced by PDA coating). Though only a visual observation, the Day 14 overlays for the conductive DBM/PCL scaffolding groups (without and with ES) appear to be overtaken by mineralized nodules stained in red (as opposed to blue-stained nuclei). This could be due to several reasons, including the effects that the enhanced electrical properties of the scaffolds have on differentiating cells. These groups, particularly the group exposed to ES, also appear to display larger chunks of stained mineral (shown in Figure 9 inserts) compared to PCL-only and nonconductive DBM-infused scaffolding groups.

## 4 Conclusions

Recently, approaches to engineer bioactive scaffolding for bone-like tissues have evolved with the use of conductive materials in place of free-releasing growth factors. These materials have been shown to be useful in tissue engineering applications, particularly when combined with exogenous ES. This study attempts to further exploit these regenerative potentials by incorporating bioactive DBM directly into the scaffolding matrix of a 3D-printed synthetic scaffold with improved electrical properties. Altogether, our results show that the methods used here are capable of (1) easily producing bioactive conductive scaffolds with controllable geometries (i.e., 3D-printed) and mechanical properties appropriate for bone tissue engineering, (2) tailoring the electrical properties of scaffolds postfabrication to suit particular regenerative needs (i.e., cell attachment and growth), and (3) enhancing osteogenic differentiation of hMSCs in vitro. The scaffolds formed in this study more closely mimic natural bone tissue, having both improved biological and electrical properties over those of currently used synthetic bone scaffolds. Therefore, 3D-printed DBM-based conductive scaffolds combined with ES are suitable for bone tissue engineering. However, further studies should be conducted using these materials in vitro before in vivo models can be investigated.

## Supporting Information Available

Detailed methods for producing DBM powder, printing inks used for fabricating PCL and DBM/PCL composite scaffolds, SEM analysis of scaffolds, and FTIR spectra of PCL and PDA-coated PCL scaffolds (PDF)
