# Supplementary material for: 3D-Printed Demineralized Bone Matrix-Based Conductive Scaffolds Combined with Electrical Stimulation for Bone Tissue Engineering Applications
Source: ACS Appl Bio Mater. 2024 Jun 21;7(7):4366–78. doi: 10.1021/acsabm.4c00236 (PMC11253088; doi:10.1021/acsabm.4c00236)
Supplement: Supplementary file 1 — mt4c00236_si_001.pdf [file mt4c00236_si_001.pdf]

Supporting Information

3D-Printed Demineralized Bone Matrix-Based Conductive Scaffolds Combined with Electrical Stimulation for Bone Tissue Engineering Applications

Damion T. Dixon[1], Erika N. Landree[2], and Cheryl T. Gomillion[2,]*

[1]School of Environmental, Civil, Agricultural and Mechanical Engineering, College of Engineering, University of Georgia, Athens, Georgia 30602, United States

[2]School of Chemical, Materials and Biomedical Engineering, College of Engineering, University of Georgia, Athens, Georgia 30602, United States

Corresponding Author:

Prof. Cheryl T. Gomillion, Ph.D.

Associate Professor

University of Georgia

Athens, GA 30602

Email: ctgomillion@uga.edu

1. SUPPORTING METHODS

1.1 Preparation of Demineralized Bone Matrix (DBM) Powder

Human femurs were provided by MTF Biologics (Edison, NJ, USA) through their Non-Transplantable Tissue Program. Diaphyseal cortical/cancellous sections of the frozen femurs were first prepared using a chop saw (DeWalt, Towson, MD, USA) before being further segmented into roughly 1 cm x 1 cm x 1 cm blocks using a Dremel tool (Dremel, Racine, WI, USA). Bone fragments were then placed into a defatting solution consisting of a 3:1 volume ratio of chloroform to methanol and constantly stirred (700 rpm) for approximately 4 hours. After defatting, bone fragments were washed in one volume of 100% ethanol (KOPTEC, 200 proof) for 30 minutes under constant stirring; the ethanol was removed, and the samples were left to dry overnight (~12 hours) at 4°C. After refreezing at -80°C, samples were ground into a uniform powder (< 125 µm) using a Micro-Mill II Grinder (Bel-Art, Warminster, PA, USA) followed by demineralization in ten volumes of 0.5 *N* hydrochloric acid (HCl) using an established lab protocol. Demineralization was confirmed by performing a calcium oxalate test (Pointe Scientific, Canton, MI, USA) on the spent HCl from each demineralization cycle. After two 30-minute cycles of demineralization, the DBM powder was washed in one volume of 100% ethanol for 30 minutes under constant stirring; the ethanol was removed, and samples were left to dry for roughly 6 hours at 4°C. The DBM powder was stored at -80°C until further use. Figure S1 provides an overview of the methods used for DBM powder preparation.

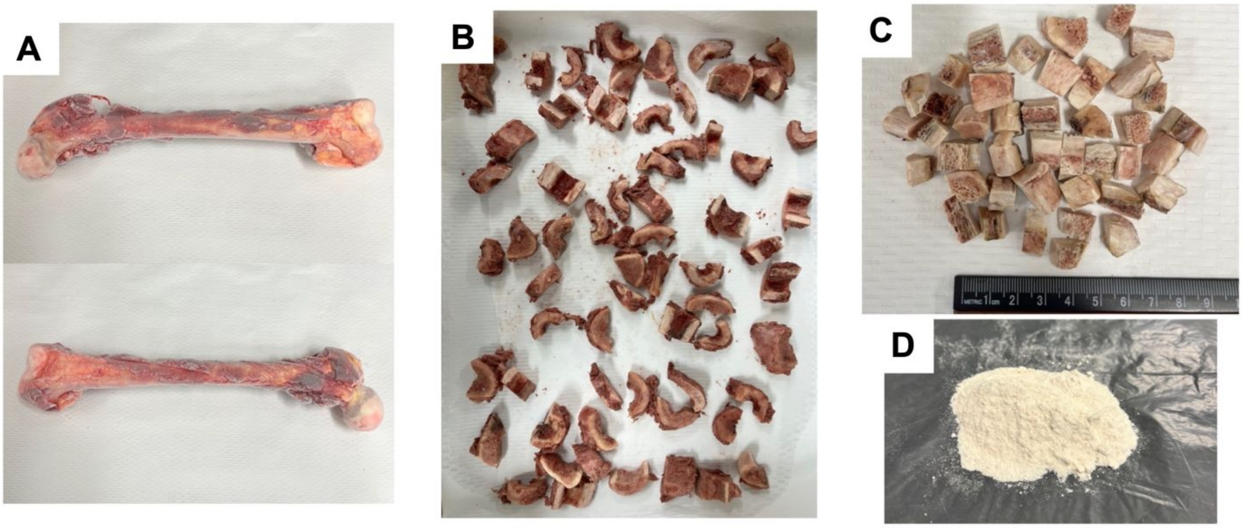

Figure S1. Overview of methods used to prepare DBM powder. (A) Frozen human femurs received from MTF Biologics. (B) Diaphyseal cortical/cancellous sections before cleaning. (C) Cortical/cancellous bone blocks after defatting. (D) Demineralized bone matrix powder (< 125 μm) used to formulate printing inks for composite scaffolds.

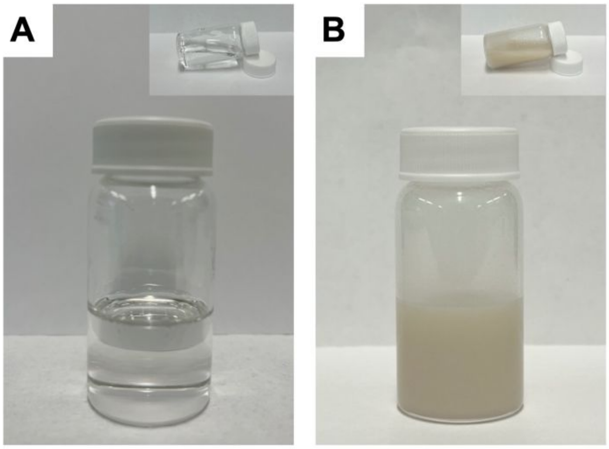

Figure S2. Inks used to print (A) PCL and (B) DBM/PCL composite scaffolds.

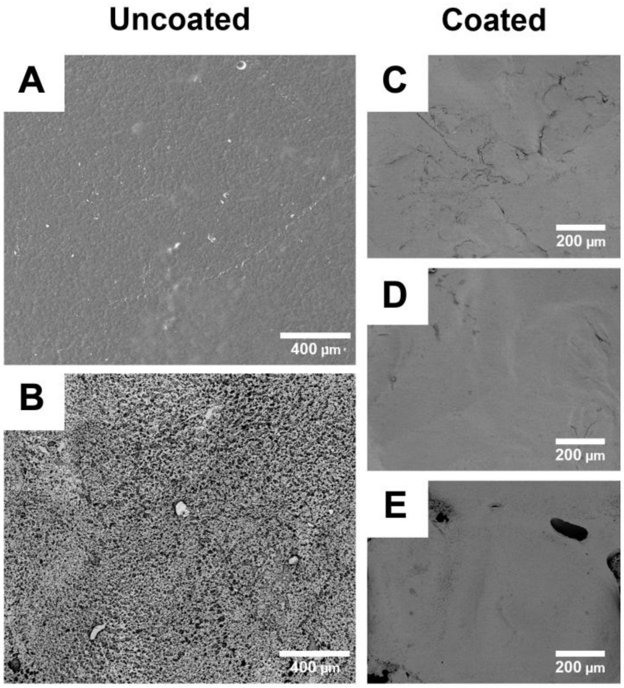

Figure S3. SEM images of (A) PCL, (B) DBM/PCL, and (C through E) DBM/PCL composite scaffolds coated with various dilutions of a conductive polymer coating (C, 1%; D, 10%; E, 20%).

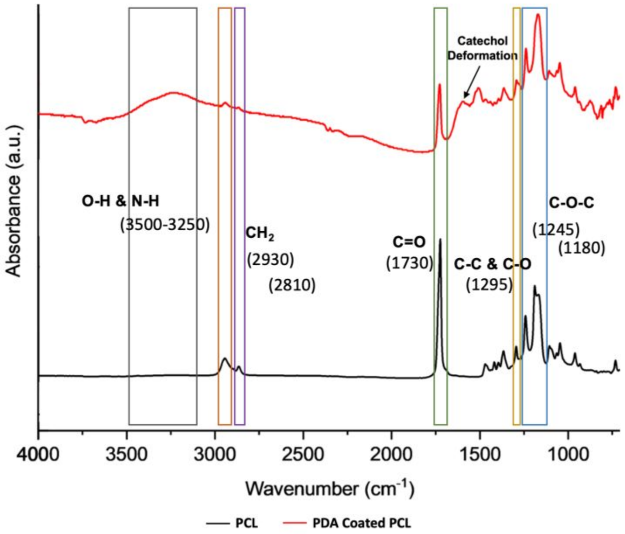

Figure S4. FTIR spectra of PCL and PDA coated PCL scaffolds confirming the self-polymerization of polydopamine onto PCL scaffolds.
